# 超高效液相色谱-三重四极杆质谱法测定地表水和废水中8种紫外线吸收剂

**DOI:** 10.3724/SP.J.1123.2025.08001

**Published:** 2026-04-08

**Authors:** Huijing SUN, Beibei ZHANG, Mengqiao HUANG, Hui WANG, Guanjiu HU, Huimin CHEN

**Affiliations:** 1.江苏省环境监测中心，国家环境保护地表水环境有机污染物监测分析重点实验室，江苏 南京 210019; 1. Jiangsu Provincial Environmental Monitoring Center，State Environmental Protection Key Laboratory of Monitoring and Analysis for Organic Pollutants in Surface Water，Nanjing 210019，China; 2.江苏省淮安环境监测中心，江苏 淮安 223001; 2. Huai’an Environmental Monitoring Center of Jiangsu Provincial，Huai’an 223001，China; 3.上海爱博才思分析仪器贸易有限公司，上海 200335; 3. SCIEX Analytical Instrument Trading Co，Shanghai 200335，China

**Keywords:** 紫外线吸收剂, 超高效液相色谱-三重四极杆质谱, 液液萃取, ultraviolet （UV） absorbers, ultra performance liquid chromatography-tandem mass spectrometry （UPLC-MS/MS）, liquid-liquid extraction （LLE）

## Abstract

紫外线吸收剂是一种广泛应用于工业的光稳定剂，具有高持久性和生物蓄积性，难以通过自然方式降解，可能长期残留在环境中，并通过食物链累积，对生态系统和生物多样性造成不可逆的损害。本研究建立了一种基于液液萃取-超高效液相色谱-三重四极杆质谱测定地表水和废水中8种紫外线吸收剂的方法。量取100 mL水样，采用二氯甲烷液液萃取，氮吹浓缩，甲醇复溶，加入内标混匀后，通过BEH C18色谱柱（100 mm×2.1 mm，1.7 μm）分离，以0.2%甲酸水溶液-乙腈为流动相进行梯度洗脱，多反应监测（MRM）模式检测，内标法定量。8种紫外线吸收剂在1~100 ng/mL范围内线性良好，方法检出限为1.3～2.8 ng/L，方法定量限为5.2～11.2 ng/L，在低、中、高3个加标水平下的回收率为80.3%～117.8%，相对标准偏差为1.4%～10.5%。将该方法应用于10份印染废水样品的检测，废水中共检出UV-329、UV-326、UV-328、UV-350这4种紫外线吸收剂，其中UV-329检出率最高，且检出的质量浓度占比高达85%，检出质量浓度范围为5.2~2 109 ng/L。该方法灵敏度好，准确度高，可应用于地表水和废水中紫外线吸收剂的检测。

## Abstract (en)

Ultraviolet （UV） absorbers are a group of chemicals widely used in various industrial and consumer products， such as plastics， coatings， and personal care products， to protect against UV radiation. Among them， benzotriazole derivatives （e.g. UV-326， UV-327， UV-328， UV-329， and UV-P） are the most frequently employed. Owing to their widespread use and potential persistence in the environment， these compounds have been detected in various environmental matrices， including surface water， wastewater， sediment， and biota. Certain UV stabilizers have been reported to exhibit endocrine-disrupting properties and pose potential ecological risks. Therefore， developing sensitive and reliable analytical methods for monitoring these compounds in environmental samples is essential. To address the need for reliable detection methods， this study developed a robust method based on liquid-liquid extraction （LLE） coupled with ultra performance liquid chromatography-triple quadrupole mass spectrometry （UPLC-MS/MS） for the simultaneous determination of eight UV absorbers in surface water and wastewater. Critical optimization of the pretreatment process focused on solvent selection and purification parameters. The finalized protocol involved extracting 100 mL water samples twice with dichloromethane. After nitrogen-assisted solvent evaporation， the residue was reconstituted in methanol and mixed with the internal standard solution. UPLC-MS/MS parameters were optimized to achieve optimal instrumental performance. The separation of the eight UVs was performed using a BEH C18 column （100 mm × 2.1 mm， 1.7 μm） with a gradient elution system consisting of 0.2% （mass fraction） formic acid aqueous solution and acetonitrile at a flow rate of 0.4 mL/min. The injection volume was 2 μL. Detection was performed in positive ion mode using multiple reaction monitoring （MRM）， with an electrospray ionization voltage set at 5 500 V. Quantification was achieved via internal standard calibration to ensure precision and accuracy. The method demonstrated excellent linearity for all target compounds across their respective concentration ranges， with a correlation coefficient （*r*）>0.995. The method detection limits （MDLs） ranged from 1.3 ng/L to 2.8 ng/L， indicating high sensitivity. Recovery tests conducted at low， medium， and high spiking levels （20， 200， and 800 ng/L） yielded recoveries of 80.3%-117.8%， with relative standard deviations （RSDs） of 1.4%–10.5%， confirming the method’s robustness across different sample matrices. Application of the method to 10 textile dyeing wastewater samples revealed the presence of four UV absorbers： UV-329， UV-326， UV-328， and UV-350. Notably， UV-329 showed the highest detection frequency and accounted for 85% of the total detected mass concentrations， ranging from 5.2 to 2 109 ng/L. Its prevalence suggests its widespread use in industrial processes and potential persistence in aquatic environments. In conclusion， the developed method is highly sensitive， accurate， and reliable for detecting UV absorbers in environmental water samples. Its successful application to surface water and wastewater analysis provides a valuable tool for monitoring these emerging contaminants， thereby supporting the assessment of their environmental and health risks. This study highlights the importance of continued monitoring and regulation of UV absorbers to mitigate their potential adverse effects on ecosystems and human health.

紫外线吸收剂（ultraviolet absorbers， UVs）是一类基于高度共轭结构实现光稳定功能的功能性化合物，其作用机制为选择性吸收特定波长紫外线辐射，并通过能量转化以热能或其他无害形式释放，从而保持自身化学稳定性^［1-3］^，根据其用途，紫外线吸收剂可分为紫外线稳定剂和紫外线过滤剂^［4，5］^。其中，紫外线稳定剂作为功能添加剂广泛应用于工业制品及日常消费品领域，包括塑料制品、涂料、黏合剂、纺织品及家具等^［5-10］^，通过抑制紫外线引发的光降解与氧化反应，显著延长材料服役寿命。而紫外线过滤剂则作为关键活性成分添加于防晒霜等个人护理产品中，以实现对人体皮肤的紫外线防护功能^［8，11-13］^。近年环境监测数据显示，UVs已在全球范围内的水体、大气颗粒物及沉积物中广泛检出，其环境残留浓度普遍处于ng/L至μg/L量级^［14-19］^。鉴于多数UVs兼具光化学稳定性与亲脂性特征，其在环境介质中难以降解且易通过食物链产生生物蓄积效应。毒理学研究表明，此类化合物具有内分泌干扰、雌激素活性及生殖发育毒性等潜在风险^［20，21］^，且其毒性效应可能随化合物亲脂性增强而加剧，进而对生态系统与人体健康构成威胁^［22］^。

鉴于UVs的环境风险，全球多国已逐步强化其使用管控。我国生态环境部于2023年颁布《第一批化学物质环境风险优先评估计划》（环办固体32号），将2-（2′-羟基-3′，5′-二叔丁基苯基）-苯并三唑（UV-320）与2-（2′-羟基-3′，5′-二叔丁基苯基）-5-氯代苯并三唑（UV-327）纳入优先评估名录。日本早在2004年通过《化学物质控制法》将苯并三唑类UVs列为一级监控物质，并于2006年单独增补UV-320至该名录。欧盟REACH法规亦于2014年将UV-320和2-［2-羟基-3，5-二（1，1-二甲基丙基苯基）］-2*H*-苯并三唑（UV-328）纳入高关注物质（SVHC）清单，限定其含量不得超过0.1%，2015年进一步将UV-327与2-（2′-羟基-3′-异丁基-5′-叔丁基苯基）苯并三唑（UV-350）列入第14批SVHC名录。此外，OekoText Standard 100（2016版）对生态纺织品中UV-320、UV-327、UV-328及UV-350的质量分数设置了0.1%的限值要求。

现有UVs检测技术主要为气相色谱-质谱联用（GC-MS）^［23-29］^和液相色谱-质谱联用（LC-MS）^［5，10，14，21，30-34］^_。_紫外线吸收剂的检测方法发展趋势如下：在2005-2010年，紫外线吸收剂的测定以GC-MS为主；2011-2015年，LC-MS/MS和GC-MS 两者在数量上相当；2016年之后，LC-MS/MS占绝对优势，其方法检出限普遍处于ng/L至μg/L水平。目前尚无涉及环境水体中紫外线吸收剂的国家标准、行业标准及地方标准，已颁布实施的国家标准、行业标准及地方标准多用于纺织业、涂料等的紫外线吸收剂含量检测。本研究基于液液萃取（LLE）-超高效液相色谱-三重四极杆质谱（UPLC-MS/MS）联用技术，通过系统优化萃取流程和净化流程，建立了适用于地表水和废水中UVs的高灵敏度检测方法。该方法兼具优异的选择性与抗基质干扰能力，可为准确获取UVs的环境赋存特征及开展生态风险评估提供可靠技术支撑。

## 1 实验部分

### 1.1 仪器和试剂

超高效液相色谱-三重四极杆质谱联用仪（SCIEX Triple Quad 6500，美国Sciex公司）；0.22 μm聚四氟乙烯滤膜（美国Agilent公司）；实验用水为Milli-Q水（美国Millipore公司）；弗罗里硅土小柱（1 000 mg/6 mL；美国Agilent公司）。

甲醇、乙腈、甲酸、二氯甲烷（色谱纯，美国Merck公司）；无水硫酸钠、氯化钠（德国CNW公司）。

8种紫外线吸收剂包括UV-320、UV-327、2-（2′-羟基-5′-甲基苯基）苯并三唑（UV-P）、2′-（2′-羟基-3′-叔丁基-5′-甲基苯基）-5-氯苯并三唑（UV-326）、UV-328、2-（2′-羟基-5′-叔辛基苯基）苯并三唑（UV-329）、2-（5-叔丁基-2-羟苯基）苯并三唑（UV-PS）、UV-350，均购自上海安谱实验科技有限公司，纯度均大于98.0%。

8种内标包括UV326-d_3_、UV327-d_20_、UV328-d_12_、UV329-^13^C_6_、UV-P-d_3_、UV-350-d_4_、UV-320-d_4_、UV-PS-d_9_，均购自德国Dr．Ehrenstorfer公司，纯度均大于98.0%。

### 1.2 标准溶液配制

标准品用甲醇配制成1 000 μg/mL的标准储备液，于-20 ℃冰箱保存。使用时，取适量储备液，用甲醇稀释配制成质量浓度为1 μg/mL的混合标准中间液，再根据需要用甲醇稀释至所需浓度。

8种内标分别用甲醇配制成100 μg/mL的内标储备液；用甲醇进一步稀释配制质量浓度为2.5 μg/mL的内标混合使用液。

### 1.3 样品前处理

量取100 mL水样至分液漏斗中，用氢氧化钠溶液或盐酸溶液调节pH值至5~10，加入3.0 g氯化钠，移取10 mL二氯甲烷于分液漏斗中，振摇10 min后静置分层5 min，收集下层有机相，再加入10 mL二氯甲烷重复萃取一次，收集有机相，合并两次萃取液，经无水硫酸钠干燥。

地表水等基质较为干净的样品：萃取液直接经氮气吹干，加入1.0 mL甲醇复溶，最后加入内标使用液10 μL，混匀后过滤膜，进样分析。

工业废水等基质复杂的样品需进行如下净化：将萃取液氮吹至干，加入1 mL正己烷复溶，待净化。先用10 mL正己烷活化弗罗里硅土小柱，在填料暴露于空气之前，关闭控制阀，弃去流出液。将浓缩后的提取液转移至弗罗里硅土小柱中，用1 mL正己烷洗涤浓缩器皿，洗液全部转移至小柱中，收集流出液，加入4 mL正己烷-二氯甲烷（1∶1，体积比）进行洗脱，浸润2 min，缓慢打开控制阀，继续加入4 mL正己烷-二氯甲烷（1∶1，体积比），接收全部洗脱液。洗脱液浓缩至近干后，加入1.0 mL甲醇复溶，加入内标使用液10 μL，混匀后过滤膜，进样分析。

### 1.4 分析条件

#### 1.4.1 色谱条件

色谱柱：BEH C18柱（100 mm×2.1 mm，1.7 μm，美国沃特世公司）；流动相A为0.2%（体积分数）甲酸水溶液，流动相B为乙腈；流速：0.4 mL/min；梯度洗脱程序：0~1.5 min，10%B；1.5~3.0 min，10%B~65%B；3.0~7.0 min，65%B~90%B；7.0~15.0 min，90%B；15.0~15.1 min，90%B~10%B；15.1~18.0 min，10%B。进样量：2 μL。

#### 1.4.2 质谱条件

采用电喷雾电离（ESI）源，离子源加热温度为250 ℃，检测方式为多反应监测（MRM）模式。正离子检测。喷雾电压为5 500 V；雾化气压力为345 kPa （50 psi）；辅助气压力为345 kPa （50 psi）；气帘气压力为207 kPa （30 psi）。各化合物的质谱参数见表1。

**表1:** 8种紫外线吸收剂及内标的质谱参数

Compound	Name	Parent ion （*m/z*）	Daughter ion （*m/z*）	Declustering potential/V	Collison energy/eV	IS
2-（Benzotriazol-2-yl）-4，6-ditert-butylphenol	UV-320	324.2	268.3^*^	130	32	UV320-d_4_
		324.2	212.3	130	39	
2，4-Ditert-butyl-6-（5-chlorobenzo-triazol-2-yl）phenol	UV-327	358.2	302.2^*^	120	31	UV327-d_20_
		360.2	304.0	120	31	
2-（Benzotriazol-2-yl）-4-methylphenol	UV-P	226.0	120.0^*^	75	25	UV-P-d_3_
		226.0	107.0	75	28	
2-（5-*tert*-Butyl-2-hydroxyphenyl）-benzotriazole	UV-PS	268.0	212.1^*^	120	29	UV-PS-d_9_
		268.0	166.0	120	40	
2-*tert*-Butyl-6-（5-chlorobenzotriazol-2-yl）-4-methylphenol	UV-326	316.2	260.1^*^	137	28	UV326-d_3_
		316.2	154.2	137	33	
2-（2*H*-Benzotriazol-2-yl）-4，6-di-*tert*-pentylphenol	UV-328	352.2	282.2^*^	100	32	UV328-d_12_
		352.2	212.3	100	39	
2-（2*H*-Benzotriazole-2-yl）-4-（1，1，3，3-tetramethylbutyl）phenol	UV-329	324.2	212.0^*^	80	37	UV329-^13^C_6_
		324.2	57.0	80	60	
2-（3-*sec*-Butyl-5-*tert*-butyl-2-hydroxy-phenyl）benzotriazole	UV-350	324.2	268.1^*^	80	30	UV350-d_4_
		324.2	212.2	80	39	
	UV-P-d_3_	229.2	121.3	75	25	
	UV-327-d_20_	378.3	314.2	120	31	
	UV-328-d_12_	364.3	289.3	100	32	
	UV-329-^13^C_6_	330.2	218.0	80	37	
	UV-326-d_3_	319.2	263.0	137	33	
	UV-320-d_4_	328.2	272.3	130	32	
	UV350-d_4_	328.2	272.3	80	30	
	UV-PS-d_9_	277.0	214.0	120	29	

* Quantitative ion.

## 2 结果与讨论

### 2.1 质谱条件的优化

配制8种紫外线吸收剂及其内标混合溶液（100 μg/L），在ESI源和正离子扫描模式下采用流动注射进入质谱进行扫描，确定最佳去簇电压、碰撞能量及各化合物的母离子和子离子等质谱参数，优化后的质谱条件见表1。

### 2.2 色谱条件的优化

在ESI^+^模式下，为了保证化合物更好地质子化，实验比对了含5 mmol/L甲酸铵的0.1%甲酸水溶液-乙腈和0.2%甲酸水溶液-乙腈作为流动相对目标物灵敏度和分离度的影响，结果表明（见图1），以0.2%甲酸水溶液-乙腈作为流动相时，化合物的响应更好，这是由于更高的甲酸浓度提供了更充足的质子H^+^，提高了质子化效率，因此最终选择0.2%甲酸水溶液-乙腈作为流动相。

**图1:**
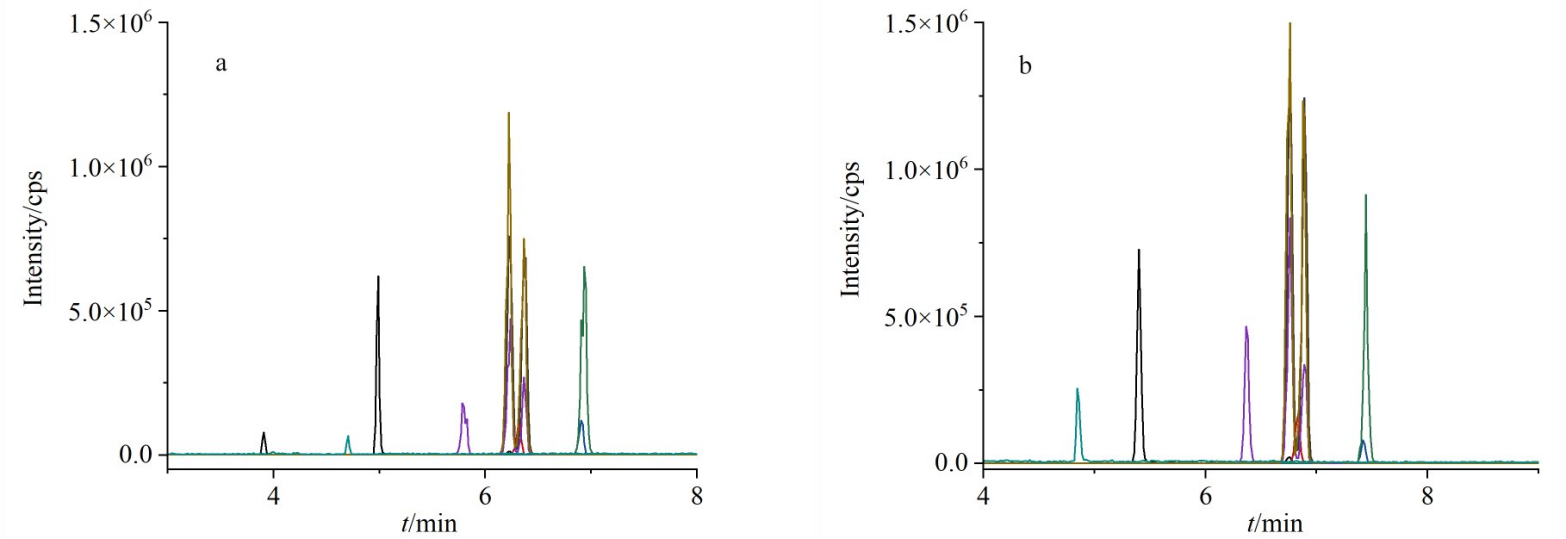
不同流动相条件下8种紫外线吸收剂的色谱图

### 2.3 水样pH的选择

为考察8种紫外线吸收剂在不同pH值下的萃取回收率，比对了空白加标样品在pH 3~10时各目标化合物的回收效果。结果如图2所示，当pH=3时，各目标物的回收率范围为55.6%~113.7%；当pH=5~10时，各目标物的回收率为71.6%~103.3%。化合物中含有苯并三唑环属于碱性基团，在中性至弱碱性条件下，目标化合物以疏水性的中性分子形态存在，更易于被有机溶剂萃取，而在酸性条件下其碱性基团被质子化形成亲水性的阳离子，导致回收率降低，故紫外线吸收剂更适合在中性至弱碱性条件下富集。因此，本实验选择在pH 5~10范围内进行萃取。

**图2:**
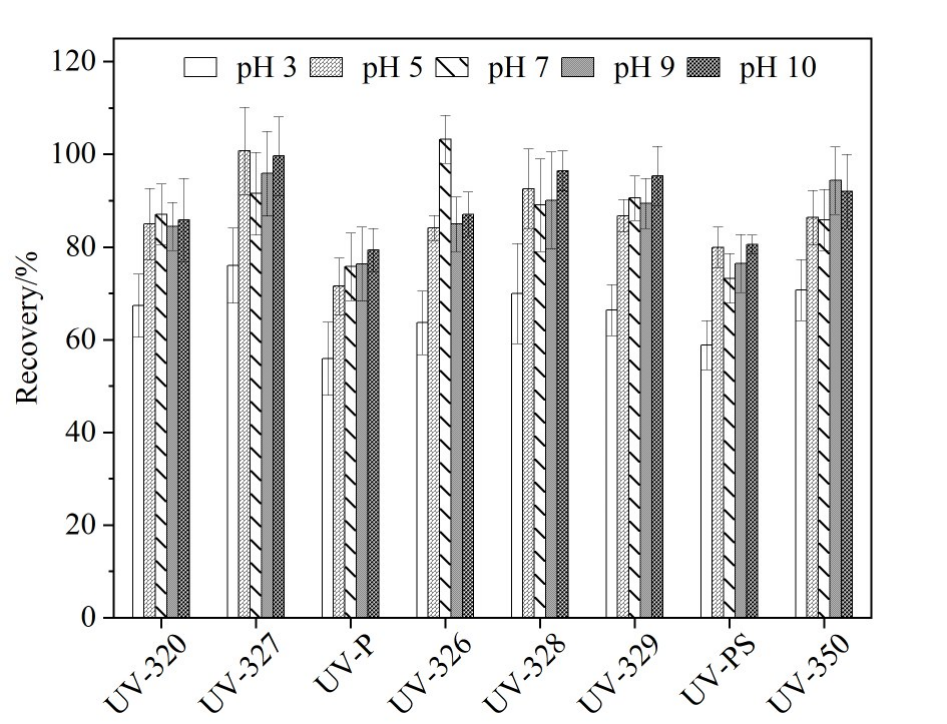
不同pH值对8种紫外线吸收剂回收率的影响（*n*=6）

### 2.4 萃取溶剂的选择

为了考察不同萃取溶剂对8种紫外线吸收剂的萃取效果，对比了乙酸乙酯、正己烷、二氯甲烷对各组分回收率的影响。量取空白纯水100 mL，加标量80 ng，各组分在3种萃取溶剂中的回收率结果见图3。用乙酸乙酯、正己烷和二氯甲烷作为萃取溶剂时8种化合物的回收率都≥70%。

**图3:**
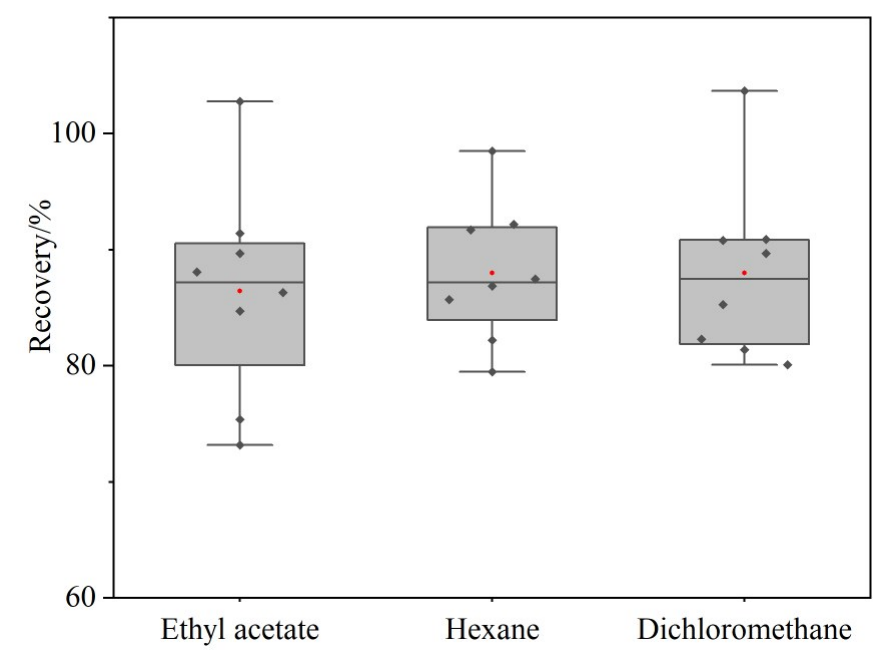
采用3种萃取溶剂时8种紫外线吸收剂的回收率

乙酸乙酯具有一定的水溶性，作为萃取溶剂时提取液体积变化较大，不利于分层。正己烷比重小于水，分离操作不易，多次萃取时操作烦琐。二氯甲烷相对乙酸乙酯沸点低，萃取后易于浓缩与溶剂转换，密度比水大，且分层明显，分液也容易操作，故最终采用二氯甲烷为萃取溶剂。

### 2.5 净化条件的选择

废水样品基质组成复杂，如不经过净化会对目标化合物产生基质效应，从而影响定量的准确性。印染废水具有基质复杂、色度高、含有大量的有机物和金属离子^［35］^的特点，因此将其选定为净化试验的实际样品。比较印染废水未净化及采用弗罗里硅土小柱净化时，以正己烷-二氯甲烷（1∶1，体积比）、丙酮-正己烷（1∶1，体积比）、正己烷-二氯甲烷（9∶1，体积比）分别作为洗脱溶剂进行洗脱后8种目标化合物回收率的差异：如图4所示，向100 mL印染废水中加入8种紫外线吸收剂，当样品未净化时，各化合物的回收率为23.5%~42.4%；当弗罗里硅土小柱作为净化柱，用正己烷-二氯甲烷（1∶1，体积比）进行洗脱时回收率为77.1%~97.1%，用丙酮-正己烷（1∶1，体积比）进行洗脱时回收率为47.6%~75.1%；用正己烷-二氯甲烷（9∶1，体积比）进行洗脱时回收率为58.8%~88.0%。由此可见，印染废水样品经过净化后，各目标化合物的回收率都得到不同程度的提升，从整体回收率结果来看，用正己烷-二氯甲烷进行洗脱效果最佳，8种紫外线吸收剂的回收率为77.1%~97.1%。

**图4:**
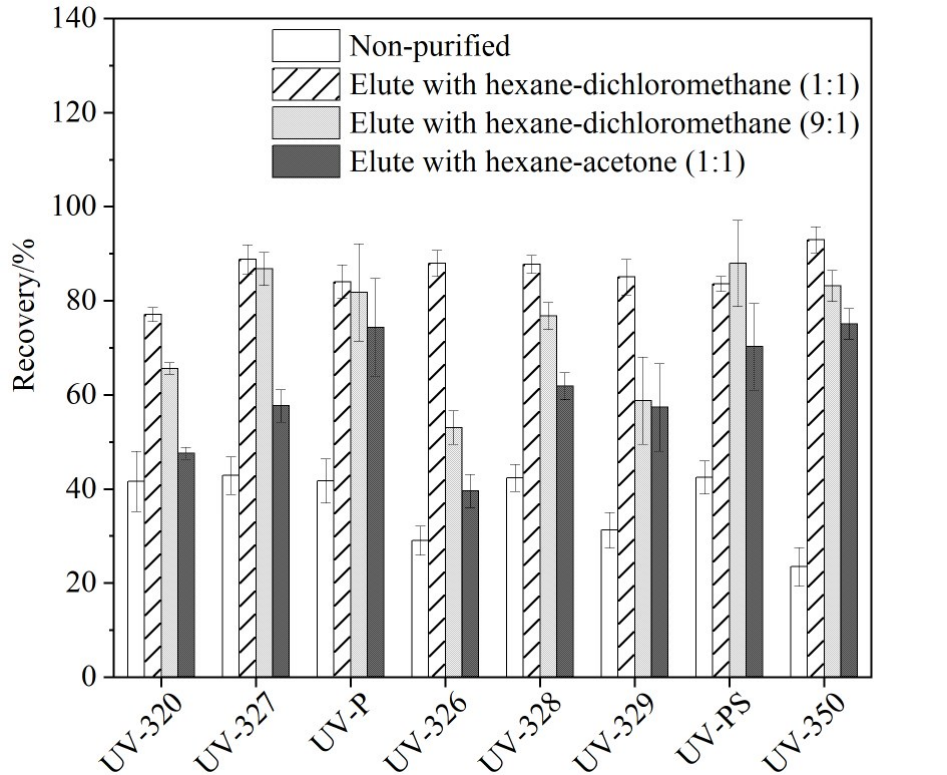
未净化以及采用3种洗脱溶剂时8种紫外线吸收剂的回收率（*n*=3）

### 2.6 方法学验证

#### 2.6.1 线性范围、方法检出限及定量限

配制系列质量浓度的混合标准溶液，按确定的分析条件进行测定，以各物质的质量浓度（ng/mL）为横坐标*X*，以其对应的峰面积与内标峰面积比值为纵坐标（*Y*），绘制标准曲线。实验结果显示，8种紫外线吸收剂在1~100 ng/mL范围内具有良好的线性关系（见表2）。

**表 2:** 8种紫外线吸收剂的回归方程、线性范围、相关系数、方法检出限、方法定量限、回收率和相对标准偏差（*n*=6）

Compound	Regression equation	*r*	MDL/（ng/L）	MQL/（ng/L）	Recovery/%	RSD/%
UV-320	*Y*=5.07×10^-1^ *X*+2.55×10^-2^	0.995	2.8	11.2	85.5-92.0	4.7-7.3
UV-327	*Y*=3.68×10^-2^ *X*+7.21×10^-3^	0.996	2.8	11.2	87.5-92.8	1.4-6.2
UV-P	*Y*=3.72×10^-2^ *X*+2.45×10^-2^	0.998	2.5	10.0	81.4-87.3	2.1-8.5
UV-326	*Y*=9.00×10^-2^ *X*+8.86×10^-3^	0.996	2.2	8.8	92.4-104.0	6.3-9.3
UV-328	*Y*=3.52×10^-1^ *X*-2.80×10^-2^	0.996	2.5	10.0	90.8-117.8	2.5-5.5
UV-329	*Y*=2.31×10^-1^ *X*-7.82×10^-2^	0.997	1.6	6.4	82.3-98.4	3.1-10.5
UV-PS	*Y*=1.52×10^-1^ *X*-1.00×10^-3^	0.999	1.3	5.2	80.3-89.6	3.7-7.7
UV-350	*Y*=6.54×10^-1^ *X*-7.856×10^-2^	0.999	2.5	10.0	84.4-90.8	4.8-5.6

*Y*： ratio of peak areas of the analyte to the internal standard； *X*： mass concentration， ng/mL； linear range： 1-100 ng/mL.

根据HJ 168-2020方法检出限测定要求，配制10 ng/L的空白水加标样品，按照样品分析过程平行测定7份。结果表明，8种紫外线吸收剂的方法检出限为1.3～2.8 ng/L，方法定量限为5.2～11.2 ng/L（见表2）。

#### 2.6.2 精密度和正确度

向实验用水中分别添加适量混合标准溶液，进行低（20 ng/L）、中（200 ng/L）和高（800 ng/L）3种浓度水平的加标回收率试验，通过回收试验考察方法的正确度和精密度。结果显示，在低、中、高3个加标水平下8种紫外线吸收剂的回收率为80.3%～117.8%，相对标准偏差为1.4%～10.5%（见表2）。方法的正确度和精密度符合方法学验证要求。

### 2.7 基质效应评价

分别采用不含待测组分的废水、地表水的基质提取液以及甲醇配制低、中、高不同质量浓度的基质标准溶液和溶剂标准溶液，上机测定。用空白基质中目标分析物的峰面积*A*和甲醇中目标分析物的峰面积*B*的比值考察基质效应的大小，基质效应被定义为*A*/*B*×100%，基质效应大于100%表示基质增强，基质效应小于100%表示基质减弱^［36］^。从表3可以看出，地表水和废水中8种化合物的基质效应为83.8%~107.3%，经过净化以及8种同位素内标进行定量能够很好地消除基质效应。

**表3:** 8种紫外线吸收剂的基质效应（*n*=6）

Compound	Matrix effects/%	
	Surface water	Waste water
UV-320	94.4	83.8
UV-327	94.4	100.3
UV-P	97.8	106.2
UV-326	95.7	107.3
UV-328	97.6	99.1
UV-329	93.7	90.7
UV-PS	103.4	95.3
UV-350	97.5	88.8

### 2.8 与固相萃取法的比较

分别比较了液液萃取法与固相萃取法对8种紫外线吸收剂的萃取回收率。参考国内外文献^［37-39］^，采用HLB小柱对水样中目标物进行富集，具体流程见图5（Process 1）。平行6份水样加标试验（加标量50 ng）结果显示，固相萃取法中多数目标化合物的回收率显著低于液液萃取法（图6）。

**图5:**
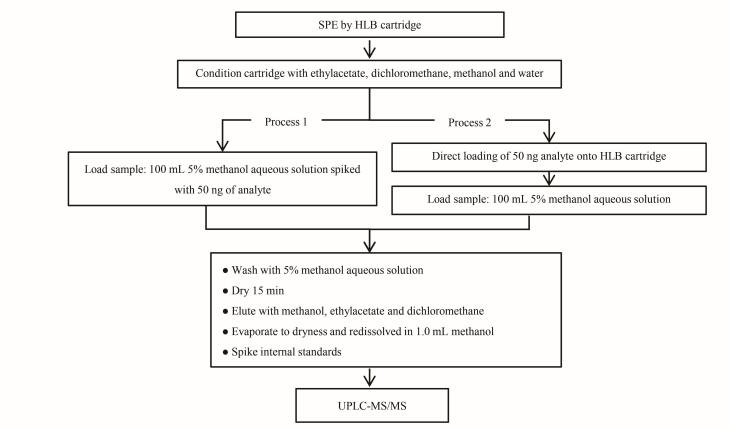
固相萃取法中两种上样流程的示意图

**图6:**
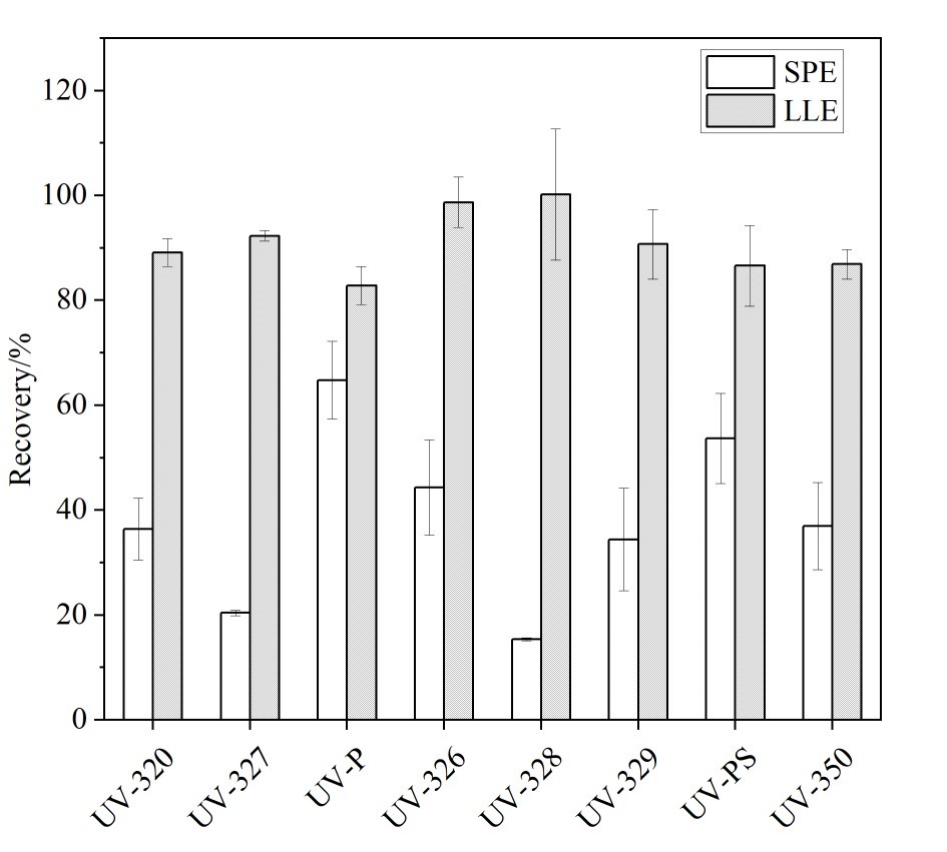
液液萃取法和固相萃取法的加标回收率（*n*=6）

为进一步探究回收率偏低的原因，我们分别采用品牌R和品牌G两款固相萃取装置，系统考察管路吸附对回收率的影响。针对每一款装置，均设计了两种上样流程进行对比：（1）将标准溶液加入100 mL空白纯水中，混匀后经固相萃取装置的管路系统加载至HLB小柱（见图5的Process 1）；（2）将标准溶液直接加至HLB小柱，随后将100 mL空白纯水经由流路加载至小柱（见图5的Process 2）。

结果（见图7）表明，两种品牌固相萃取装置经图5（Process 1）前处理后目标化合物的回收率（12.5%~64.8%）均显著低于经图5（Process 2）前处理后目标化合物的回收率（67.9%~93.7%）。这证明对于本研究中的强疏水性目标物（UV-P和UV-PS的log *K*_ow_值为3.0和4.4，其余6种化合物的log *K*_ow_值为5.5~7.3^［32］^），固相萃取上样过程中的管路吸附是导致回收率损失的一个普遍且关键的因素。理论上，为避免管路的吸附，可尝试将样品直接加载到HLB小柱上，但是HLB小柱的上样容量有限（通常<5 mL），对于体积较大的环境水样（通常为100~1 000 mL），必须依赖流路系统进行连续上样。

**图7:**
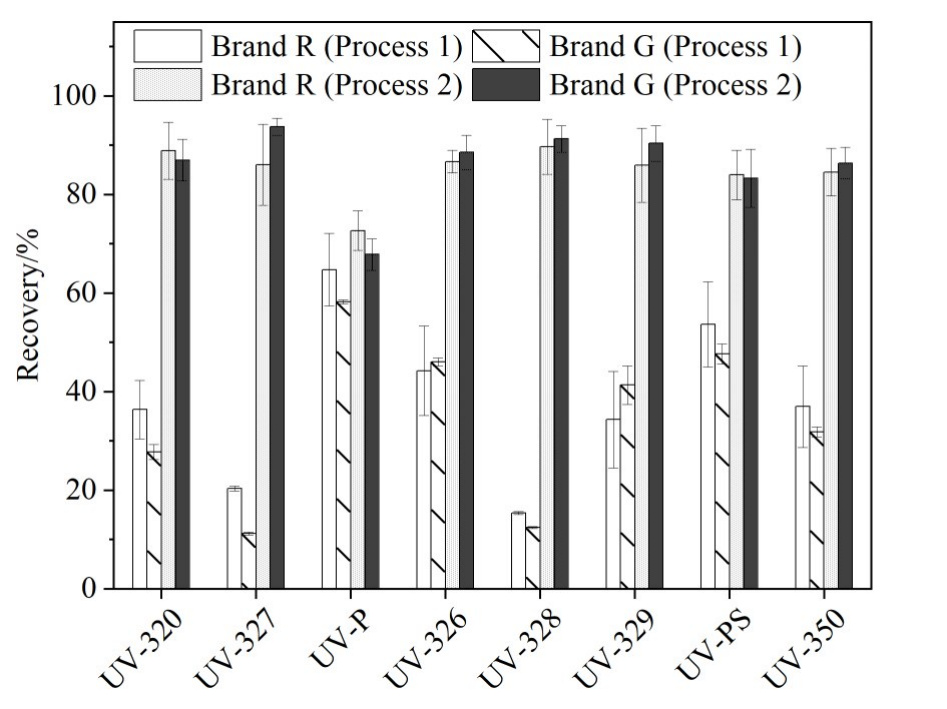
两种固相萃取装置在不同上样流程下的加标回收率比较（*n*=3）

综上，尽管固相萃取法广泛用于水体有机污染物的富集，但对于本研究所涉及的强疏水性化合物，其在现有固相萃取法中存在的管路吸附问题难以避免，且尚无简便可靠的解决方案。因此，从方法稳定性、实用性和可操作性的角度出发，二氯甲烷液液萃取法更适合于该类化合物的测定。

### 2.9 实际样品的应用

应用该方法初步分析了某印染聚集区印染厂废水中紫外线吸收剂的含量，检出的成分有UV-329、UV-326、UV-328、UV-350，其中UV-329检出率最高，且质量浓度占比高达85%，检出质量浓度为5.2~2 109 ng/L，具体数据见表4。

**表4:** 印染废水中紫外线吸收剂的含量

No.	UV-320	UV-327	UV-P	UV-326	UV-328	UV-329	UV-PS	UV-350
S01	-	-	-	-	-	-	-	-
S02	-	-	-	-	-	14.2	-	-
S03	-	-	-	196	-	163	-	-
S04	-	-	-	-	21.6	397	-	-
S05	-	-	-	-	-	109	-	-
S06	-	-	-	-	-	90.2	-	-
S07	-	-	-	-	5.2	6.3	-	-
S08	-	-	-	-	-	36.5	-	27
S09	-	-	-	-	29.3	360	-	-
S10	-	-	-	-	-	2190	-	11

-： not detected.

## 3 结论

本文建立了地表水和废水中8种紫外线吸收剂的超高效液相色谱-三重四极杆质谱测定方法。该方法灵敏度好，准确度高，能满足水中紫外线吸收剂测定的要求，可为准确掌握水体中紫外线吸收剂的污染水平及环境管理提供技术支撑。
